# Imaging glial activation in patients with post-treatment Lyme disease symptoms: a pilot study using [^11^C]DPA-713 PET

**DOI:** 10.1186/s12974-018-1381-4

**Published:** 2018-12-19

**Authors:** Jennifer M. Coughlin, Ting Yang, Alison W. Rebman, Kathleen T. Bechtold, Yong Du, William B. Mathews, Wojciech G. Lesniak, Erica A. Mihm, Sarah M. Frey, Erica S. Marshall, Hailey B. Rosenthal, Tristan A. Reekie, Michael Kassiou, Robert F. Dannals, Mark J. Soloski, John N. Aucott, Martin G. Pomper

**Affiliations:** 1 Department of Psychiatry and Behavioral Sciences, Johns Hopkins University School of Medicine, Baltimore, MD USA; 2 Russell H. Morgan Department of Radiology and Radiological Science, Johns Hopkins University School of Medicine, Baltimore, MD USA; 3 Division of Rheumatology, Department of Medicine, Johns Hopkins University School of Medicine, Baltimore, MD USA; 4 Department of Physical Medicine and Rehabilitation, Johns Hopkins University School of Medicine, Baltimore, MD USA; 5 School of Chemistry, The University of Sydney, Sydney, NSW 2006 Australia; 6 Baltimore, USA; 7 Lutherville, USA

**Keywords:** [^11^C]DPA-713, Microglia, Post-treatment Lyme disease syndrome, Neuroimaging

## Abstract

The pathophysiology of post-treatment Lyme disease syndrome (PTLDS) may be linked to overactive immunity including aberrant activity of the brain’s resident immune cells, microglia. Here we used [^11^C]DPA-713 and positron emission tomography to quantify the 18 kDa translocator protein, a marker of activated microglia or reactive astrocytes, in the brains of patients with post-treatment Lyme disease symptoms of any duration compared to healthy controls. Genotyping for the TSPO *rs6971* polymorphism was completed, and individuals with the rare, low affinity binding genotype were excluded. Data from eight brain regions demonstrated higher [^11^C]DPA-713 binding in 12 patients relative to 19 controls. [^11^C]DPA-713 PET is a promising tool to study cerebral glial activation in PTLDS and its link to cognitive symptoms.

**Electronic supplementary material:**

The online version of this article (10.1186/s12974-018-1381-4) contains supplementary material, which is available to authorized users.

## Background

Lyme disease, a vector-borne infectious disease caused by *Borrelia burgdorferi*, is endemic across large regions of the Northern Hemisphere [1]. If untreated, the bacteria can disseminate from the site of transmission (tick bite) to other organs. Prompt treatment with appropriate antibiotic therapy often leads to successful resolution of disease while lag to treatment portends worse outcome [2], including the potential for neurologic complications. Even with timely diagnosis and appropriate antibiotic treatment, 10–20% of individuals develop a constellation of symptoms that may include fatigue, pain, sleep disruption, and cognitive difficulties [3]. In contrast to “chronic Lyme disease” that is less precisely defined [4], post-treatment Lyme disease syndrome (PTLDS) is a condition characterized by symptoms that manifest within 6 months of treatment and persist 6 months or longer [3], which can be very debilitating [5].

The etiology of PTLDS remains unclear and may occur through chronic, peripheral inflammatory response with signaling factors that can diffuse across the blood-brain barrier, or through more direct central nervous system (CNS) immunity [6, 7]. Recent evidence supports persistence of non-viable spirochetes or their remnants in human joint synovial tissue after antibiotic treatment [8, 9], and rare spirochetes have been found in the CNS of antibiotic-treated monkeys [9]. In vitro models have shown that debris from treated spirochetes may elicit chronic secretion of inflammatory signaling molecules (cytokines, chemokines) by microglia, the resident immune cells in the brain, or other cell types such as oligodendrocytes or infiltrating lymphocytes [10–12]. In a recent study of primary human microglia treated with antibiotic-killed or sonicated spirochetes, the response of increased expression of inflammatory proteins was more robust than after treatment with live spirochetes [12]. Together, these data suggest that if spirochete antigens remain in the CNS after antibiotic treatment, they may facilitate a persistent neuroimmune response linked to neuropsychiatric symptoms of PTLDS. Validating this proposed pathophysiology in vivo may inform immune-modulating therapeutic strategies to improve outcomes.

The 18 kDa translocator protein (TSPO) is a mitochondrial protein that is greatly increased in its expression by activated microglia and reactive astrocytes [13]. Since cerebral TSPO can be measured using radiotracers such as [^11^C]DPA-713 with positron emission tomography (PET), imaging TSPO has proven a useful method to test for hypothesized immune activation in vivo in relevant neurological conditions such as human immunodeficiency virus-related neurodegeneration, systemic lupus erythematosus, and traumatic brain injury [14–16]. [^11^C]DPA-713 is a second-generation radiotracer for imaging TSPO that has superior binding specificity compared to the first-generation radiotracer, [^11^C]-(*R*)-PK11195 [17]. Here, we used [^11^C]DPA-713 PET in a pilot population of well-characterized patients with persistent symptoms following treatment for Lyme disease to test for high availability of cerebral TSPO relative to healthy controls. Since Lyme disease is likely to drive a systemic immune response, we posited further that the relatively high TSPO distribution in brains of these patients would be diffusely spread across brain regions rather than focal in pattern.

## Methods

### Participants

This study was conducted under a protocol approved by a Johns Hopkins Institutional Review Board and under an FDA Investigational New Drug application. All participants provided written, informed consent. Adult (age ≥ 18 years) patients were recruited from the Johns Hopkins Lyme Disease Research Center and were eligible if they had (1) a prior, medically documented case of probable or confirmed Lyme disease by CDC criteria [18], (2) history of appropriate treatment, and (3) post-treatment Lyme disease symptoms of any duration. Historical [^11^C]DPA-713 PET data from all healthy individuals 18–65 years old were pooled from two prior studies [15, 16].

Exclusion criteria for all participants included unstable health or recent infection other than Lyme disease, history of neurological condition not attributable to Lyme disease, clinical abnormality on screening assessment (blood, urine, electrocardiogram), benzodiazepine or anti-inflammatory medication use (including NSAIDs) in the past 2 weeks, substance abuse (confirmed by screening toxicology), or contraindication to imaging [magnetic resonance imaging (MRI) or PET]. Antibiotic treatment in the 2 weeks prior to imaging was allowed, with the exception of minocycline, which may affect the imaging results [19].

### Clinical and neuropsychological assessment

Among patients, the Lyme disease history was confirmed and characterized through review of medical records and a clinical interview. Every patient also completed a standardized assessment of symptoms [5] and battery of neuropsychological tests [20].

### Structural imaging

Each participant underwent a T1-weighted MRI on either a 1.5-Tesla Signa Advantage system (GE Medical Systems, Waukesha, WI, USA) or a Phillips Achieva 3 Tesla scanner (Andover, MA), to obtain a 0.8 × 0.8 × 0.8 mm^3^ three-dimensional Magnetization-Prepared Rapid Gradient-Echo sequence. MRI data were used to screen for structural abnormality and to delineate anatomical regions of interest (ROIs) after PET-MRI coregistration.

### PET acquisition

The synthesis of [^11^C]DPA-713 was conducted as previously described [21], and the product met all U.S. Pharmacopeia Convention Chapter <<823> acceptance testing criteria. Each participant was fitted with a thermoplastic face mask for head positioning and immobilization during the emission scan. After a 6-min transmission scan that was acquired prior to the emission scan, [^11^C]DPA-713 was delivered with high specific radioactivity (Table 1) via intravenous bolus injection at the beginning of a 90-min dynamic list mode PET acquisition. The average injected dose was 691.9 ± 18.3 MBq. The arterial plasma input function was acquired from 25 to 35 blood samples collected over the duration of the scan, with additional blood sample collection at eight time points for measurement of metabolites as in our prior work [22]. PET data were acquired on a High Resolution Research Tomograph (Siemens Healthcare, Knoxville, TN, USA), and the 90-min listmode data were binned into 30 frames and reconstructed using the iterative ordered subset expectation maximization algorithm, with correction for radioactive decay, dead time, attenuation, scatter and random coincidences in an identical fashion to our previous work [15, 16]. Attenuation maps were generated from the transmission data. Reconstructed images consisted of cubic voxels, each 1.22 mm^3^, with dimensions of 31 cm × 31 cm (transaxially) and 25 cm (axially).

**Table 1:** Demographic characteristics and PET parameters across the study population

	Healthy controls (*N* = 19)	Patients with persistent symptoms following treated Lyme disease (*N* = 12)	*P* ^a^
Age, years	51.0 [38.5, 56.0] (22.0, 62.0)	42.0 [39.5, 47.3] (24.0, 63.0)	0.361
Sex, male	13 (68.4%)	5 (41.7%)	0.262
Race			0.002
White	8 (42.1%)	12 (100.0%)	
Black	10 (52.6%)	0 (0.0%)	
Mixed	1 (5.3%)	0 (0.0%)	
Body mass index	27.7 ± 4.1 (20.6, 34.0)	25.8 ± 3.8 (20.8, 33.2)	0.193
Genotype, C/T	6 (31.6%)	8 (66.7%)	0.075
Injected dose (MBq)	692.3 ± 20.4	691.4 ± 15.3	0.886
Injected mass (μg)	1.0 ± 0.4	1.0 ± 0.5	0.839
Specific activity (GBq/μmol)	293.6 ± 177.8	310.4 ± 101.1	0.741
Regional volume ratio^b^			
Cerebellum	0.063 ± 0.009 (0.049, 0.080)	0.073 ± 0.005 (0.065, 0.081)	< 0.001
Hippocampus	0.006 ± 0.001 (0.005, 0.007)	0.006 ± 0.000 (0.005, 0.006)	0.363
Occipital Cortex	0.030 ± 0.003 (0.025, 0.037)	0.028 ± 0.004 (0.017, 0.033)	0.072
Frontal cortex	0.111 [0.107, 0.114] (0.085, 0.125)	0.111 [0.109, 0.114] (0.058, 0.122)	0.984
Parietal cortex	0.074 [0.067, 0.077] (0.060, 0.086)	0.074 [0.070, 0.076] (0.034, 0.078)	0.826
Thalamus	0.011 [0.010, 0.011] (0.008, 0.012)	0.010 [0.010, 0.011] (0.010, 0.014)	0.889
Temporal cortex	0.063 [0.061, 0.067] (0.050, 0.076)	0.063 [0.061, 0.065] (0.021, 0.072)	0.826
Cingulate cortex	0.013 [0.012, 0.014] (0.011, 0.015)	0.013 [0.013, 0.014] (0.003, 0.015)	0.704

Data from normally distributed continuous variables are presented as mean ± standard deviation (range) and from continuous variables not normally distributed as median [25th percentile, 75th percentile] (range). Data from categorical variables are presented as count (%). ^a^Comparison between groups were conducted using Fisher’s exact tests for categorical variables, and *t* tests or Wilcoxon rank sum tests for continuous variables as appropriate. ^b^Regional volume ratios are equivalent to the volume of each region normalized to total intracranial volume and are unitless

Use of TSPO-targeting second-generation radiotracers, including [^11^C]DPA-713, must account for the effect of a single nucleotide polymorphism (rs6971) within *TSPO* on radiotracer binding affinity [16]. This genotyping was completed for each participant, with inclusion of imaging data from individuals with high or mixed affinity binding phenotypes (C/C or C/T genotype) and exclusion of those with the low affinity phenotype (T/T genotype) [16].

### Image analysis

The software package PMOD (v3.7, PMOD Technologies Ltd., Zurich, Switzerland) was used for PET image processing and kinetic analysis. Inter-frame motion correction was performed by rigidly realigning each of the 30 frames to the 0–30-min mean PET image, the latter obtained by averaging frames 1 through 18. The 0–30-min PET mean image and then each of the 30 motion-corrected PET frames were co-registered to the subject’s T1-weighted MRI using rigid transformations. Time-activity curves were generated from PET data in each region of interest (ROI) that were segmented from the T1-weighted MRI of each participant using the FreeSurfer image analysis suite (http://surfer.nmr.mgh.harvard.edu/). Eight ROIs were selected a priori (Additional file 1: Figure S1) and included cortical (frontal, parietal, temporal, occipital, cingulate) and subcortical (hippocampus, thalamus) regions. The volume of each ROI normalized to total intracranial volume (regional volume ratio) was also calculated and used to test for regional atrophy in the patients compared to controls since this measure accounts for individual variability in head size [23].

Using the time-activity curve data, the kinetics of [^11^C]DPA-713 were modeled using Logan graphical analysis (t* = 30 min) with metabolite-corrected arterial input function from 90-min dynamic data. The model produced an estimate of total distribution volume, *V*_T_, for each region as previously validated for [^11^C]DPA-713 PET [14, 22]. V_T_ represents the ratio of radioligand concentration in brain tissue to that in plasma at equilibrium and is proportional to the receptor density in the ROI [24].

### Statistical analysis

Regional *V*_T_ values were first examined visually. Pooling data from all ROIs, regression analysis was performed on the person-region level to estimate the difference in *V*_T_ between groups, adjusting for confounding factors. Specifically, we fit a linear mixed effects regression model with *V*_T_ as the dependent variable, and group as the independent variable of interest, adjusting for *TSPO* genotype, ROI, and factors with found effects on *V*_T_ [age, body mass index (BMI)]. Person was included as a random intercept to account for the within-subject correlation of *V*_T_ across regions. The threshold for significance was set to *P* < 0.05 unless otherwise noted. Statistical analyses were performed in R 3.4.3.

## Results

The 12 patients and 19 historical controls did not significantly differ in age, gender, and BMI (Table 1), although all the patients were white and the controls varied in race. There were no differences in the injection parameters between groups. Comparison of regional volume ratios revealed no regional atrophy in patients compared to controls (Table 1).

Half of the patients had a prior confirmed case of Lyme disease, and the others had prior probable Lyme disease by CDC criteria (Table 2). Each patient reported the presence of fatigue and at least one cognitive symptom (difficulty finding words, difficulty concentrating, or memory change). The 12 patients had 17.6 ± 3.2 years of education (range 12–25 years) and their neuropsychological performance (*T*-scores) and reported symptoms are presented in Table 2.

**Table 2:** Lyme disease history and self-reported cognitive symptoms

Lyme disease history	Patients with persistent symptoms following treated Lyme disease (*N* = 12)
Medical record-confirmed Lyme disease presentation	
CDC confirmed: physician-documented erythema migrans rash^a^	6 (50.0%)
CDC probable: subjective symptoms/(+) serology^b^	6 (50.0%)
PTLDS symptom duration ≥ 6 months at study visit	8 (66.7%)
Self-reported cognitive symptoms	
Memory change, moderate or severe	7 (58.3%)
Difficulty finding words, moderate or severe	7 (58.3%)
Difficulty focusing or concentrating, moderate or severe	9 (75.0%)
Cognitive symptoms,^c^ any	12 (100.0%)
Neurocognitive test *T*-scores	
Trail making test [28], part A	52.50 ± 18.53 (17.00, 80.00)
Trail making test, part B	46.67 ± 14.25 (19.00, 68.00)
HVLT [29] total recall	48.50 ± 11.87 (32.00, 64.00)
HVLT delayed recall	47.92 ± 11.41 (25.00, 59.00)
HVLT retention	51.00 ± 9.36 (26.00, 61.00)
HVLT recognition discrimination	46.33 ± 12.52 (20.00, 58.00)
WAIS-IV [30] digit span	49.25 ± 8.28 (37.00, 67.00)
WAIS-IV coding	52.83 ± 7.72 (40.00, 70.00)
ACT [31] 9^d^	54.10 ± 7.26 (37.00, 62.00)
ACT 18^d^	54.50 ± 8.83 (38.00, 69.00)
ACT 36^d^	55.60 ± 9.92 (37.00, 69.00)
Standardized symptom questionnaires	
Fatigue Severity Scale Total Score	50.42 ± 10.33 (25.00, 63.00)
Pittsburgh Sleep Quality Index Total Score	11.40 ± 4.67 (4.00, 19.00)
Short-Form McGill Pain Questionnaire Total Score	11.67 ± 6.84 (0.00, 24.00)
Beck Depression Inventory II Total Score	14.18 ± 7.10 (3.00, 24.00)
SF-36 Physical Component Score	34.91 ± 8.24 (20.21, 50.06)
SF-36 Mental Component Score	38.60 ± 11.95 (20.86, 59.47)

*ACT* Auditory Consonant Trigrams, *HVLT* Hopkins Verbal Learning Test, *SF-36* Short-Form Health Survey, *WAIS-IV* The Wechsler Adult Intelligence Scale, 4th edition

Data from normally distributed continuous variables are presented as mean ± standard deviation (range) and from continuous variables not normally distributed as median [25th percentile, 75th percentile] (range). Data from categorical variables are presented as count (%)

^a^One patient with erythema migrans rash and Bell’s Palsy

^b^Viral-like illness/(+) ELISA/WB, or non-acute patient reported symptoms/(+) IgG-WB

^c^At least one of the following present during the past 2 weeks: (1) difficulty finding words, (2) difficulty focusing or concentrating, and (3) trouble with memory

^d^Missing data from 2 of the 12 patients (16.7%) due to the later addition of the ACT to the study protocol

**Fig. 1:**
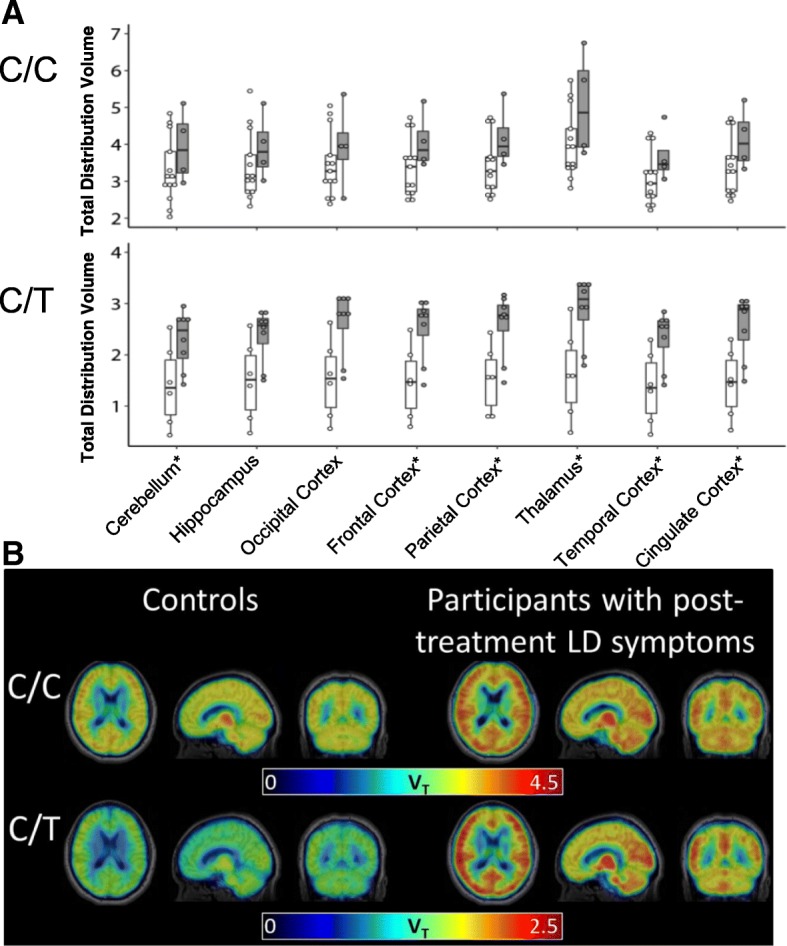
Higher [^11^C]DPA-713 regional total distribution volume (*V*_T_) values were found in 12 participants with post-treatment Lyme disease symptoms (< 6 months) or syndrome, compared to 19 healthy control participants, accounting for *TSPO* genotype (C/C vs. C/T). **a** Boxplot diagram of [^11^C]DPA-713 V_T_ across eight regions of interest from healthy controls (white data) and participants with PTLDS (gray data) that were grouped by *TSPO* genotype (C/C, upper panel; C/T, lower panel). A linear mixed effects regression model adjusting for *TSPO* genotype, brain region, and factors with found effects on binding (age, BMI) demonstrated higher *V*_T_ in the patients compared to the controls. *In secondary analyses, a regional difference was found between patients and controls using individual linear regression models fit for the region of interest and controlling for age, BMI, and genotype. These differences in individual regions did not remain significant after Bonferroni correction for the eight regions tested, which required *P* < 0.05/8 ≈ 0.006. **b** Mean parametric [^11^C]DPA-713 *V*_T_ images, displayed within each group (control, patient) in axial, sagittal, coronal views (left to right) and by *TSPO* genotype (C/C, upper panel; C/T, lower panel), demonstrate higher mean *V*_T_ in patients compared to controls. Within the study population, participants with C/C genotype included 13 controls and four patients, and those with C/T genotype included six controls and eight patients. *V*_T_ is in units of mL cm^−3^

*V*_T_ values across the eight ROIs were highly correlated among the study population (correlation coefficients *ρ* ≥ 0.97), indicating low inter-region variability within individual subjects (Additional file 2: Figure S2). Patients had higher *V*_T_ compared to controls across all ROIs (Fig. 1a). Pooling data from all ROIs, the linear mixed effects regression model adjusting for TSPO genotype, brain region, age, and BMI estimated a mean difference [95% confidence interval] of 0.58 [0.12, 1.03] between the groups (*P* = 0.015). The finding of higher *V*_T_ in the patient group relative to controls was unchanged after restricting to white participants. Additionally, we found no significant differences in *V*_T_ values when comparing CDC confirmed with probable patients, and when comparing patients with persistent symptoms lasting < 6 months after antibiotic treatment and those with PTLDS (≥ 6 months of symptoms after antibiotic treatment). Controlling for age, BMI, and genotype, individual linear regression models fit for individual ROIs showed significant differences in the cerebellum, frontal cortex, parietal cortex, thalamus, temporal cortex, and cingulate cortex. Mean parametric images of [^11^C]DPA-713 V_T_ support higher binding in patients relative to controls in other regions beyond those selected a priori (Fig. 1b).

Exploratory analyses found no relationships between [^11^C]DPA-713 *V*_T_ and performance on any cognitive test or standardized symptom score within patients after controlling for genotype, age, and BMI.

## Discussion

Our [^11^C]DPA-713 PET data are consistent with higher levels of TSPO within eight brain regions among patients with persistent symptoms following treated Lyme disease compared to healthy controls and suggest the same pattern in other regions. These findings are in keeping with previously demonstrated evidence of circulating (serum) inflammatory markers in those with PTLDS or Lyme encephalopathy [6, 25] and uniquely support immune activation in the CNS in symptomatic patients with Lyme disease post-antibiotic treatment.

Our design was rigorous in the characterization of the patient group. We included only patients with a history of CDC-confirmed or probable physician-documented Lyme disease in their medical record, appropriate antibiotic treatment, and post-treatment symptoms. Each patient in this study reported fatigue and at least one cognitive symptom. Notably, the patient sample was highly symptomatic on standardized questionnaires, and reported health-related quality of life (SF-36 scores) approximately 1.5 SD below the population mean. Future studies designed to test the relationships between [^11^C]DPA-713 binding and neuropsychological deficits or symptoms in larger, clinical samples are needed.

Our study has limitations. First, while TSPO is increased in expression by activated microglia and reactive astrocytes, the imaging signal from TSPO cannot distinguish among the diverse functional phenotypes of activated microglial cells that may contribute to detrimental or reparative effect on surrounding brain tissue [26]. Second, the observers were not blinded, which could potentially introduce subconscious bias in assessment of outcomes. Third, although our study demonstrated that patients with persistent symptoms following treatment for Lyme disease had higher levels of TSPO compared to controls, the historical control group lacked data from standardized symptom or neurocognitive assessment. Future studies, including comparative study with other control groups (such as non-infected individuals who share symptoms with the patient group) or longitudinal study of individuals who return to health after Lyme disease, will delineate further whether higher levels of TSPO are associated with persistent symptoms after treated *Borrelia burgdorferi* infection. Finally, multiple comparisons correction was not performed given the small sample size, multiple confounders, and highly correlated outcomes (correlation coefficients ρ ≥0.97).

As we demonstrate the promise of [^11^C]DPA-713 PET to study the neuroimmune response in PTLDS, we note that this imaging provides indirect measurement of TSPO. Complementary, direct study of markers of activated glia with functional phenotyping of these cells in human postmortem brain tissue or in relevant animal models will inform further these findings. Additionally, parallel assays of peripheral immune markers and longitudinal assessments of cerebral [^11^C]DPA-713 binding over the course of pre- and post-treatment Lyme disease will temporally characterize better the immune response, and comparison to data from non-infected controls with overlapping symptoms will be crucial to elucidate the links between Lyme infection, aberrant immunity, and PTLDS. These complementary data may inform whether immunotherapeutic approaches like those used in other central nervous system conditions [27] may benefit those with PTLDS. Within our growing, well-characterized clinical population, the effect of risk factors for PTLDS such as delayed time to appropriate antibiotic treatment or duration of untreated illness on [^11^C]DPA-713 binding may be probed in future work.

## Conclusions

In summary, our results support the hypothesized role of CNS immune activation in patients with PTLDS. Further study of the relationship between higher glial activation in the CNS, systemic inflammatory signaling, and cognitive performance in PTLDS is needed.

## Additional files

Additional file 1: **Figure S1.** Representative volumetric segmentation demonstrating the eight regions of interest. Abbreviations: CB, cerebellum; Th, thalamus; Hp, hippocampus; TC, temporal cortex; OC, occipital cortex; CIN, cingulate cortex; FC, frontal cortex; PC, parietal cortex. (PDF 231 kb)

Additional file 2: **Figure S2.** Correlation matrix using [^11^C]DPA-713 binding data from the eight regions of interest. All correlation coefficients are greater than or equal to 0.97 (indicating very strong correlations). Abbreviations: cb, cerebellum; hp., hippocampus; oc, occipital cortex; fc, frontal cortex; pc, parietal cortex; th, thalamus, tc, temporal cortex; cc, cingulate cortex. (PDF 233 kb)

## Abbreviations

ACT: Auditory Consonant Trigrams
BMI: Body mass index
CNS: Central nervous system
HVLT: Hopkins Verbal Learning Test
MRI: Magnetic resonance imaging
PET: Positron emission tomography
PTLDS: Post-treatment Lyme disease syndrome
ROI: Region of interest
SF-36: Short-Form Health Survey
TSPO: 18 kDa translocator protein
WAIS-IV: The Wechsler Adult Intelligence Scale, 4th edition

## References

[1] Steere AC, Strle F, Wormser GP, Hu LT, Branda JA, Hovius JW (2016). Lyme borreliosis. Nat Rev Dis Primers.

[2] Shadick NA, Phillips CB, Logigian EL, Steere AC, Kaplan RF, Berardi VP (1994). The long-term clinical outcomes of Lyme disease. A population-based retrospective cohort study. Ann Intern Med.

[3] Aucott JN (2015). Posttreatment Lyme disease syndrome. Infect Dis Clin N Am.

[4] Feder HM, Johnson BJ, O'Connell S, Shapiro ED, Steere AC, Wormser GP (2007). A critical appraisal of “chronic Lyme disease”. N Engl J Med.

[5] Rebman AW, Bechtold KT, Yang T, Mihm EA, Soloski MJ, Novak CB (2017). The clinical, symptom, and quality-of-life characterization of a well-defined group of patients with posttreatment Lyme disease syndrome. Front Med (Lausanne).

[6] Aucott JN, Soloski MJ, Rebman AW, Crowder LA, Lahey LJ, Wagner CA (2016). CCL19 as a chemokine risk factor for posttreatment Lyme disease syndrome: a prospective clinical cohort study. Clin Vaccine Immunol.

[7] Halperin JJ (2017). Neuroborreliosis. J Neurol.

[8] Steere AC, Duray PH, Butcher EC (1988). Spirochetal antigens and lymphoid cell surface markers in Lyme synovitis. Comparison with rheumatoid synovium and tonsillar lymphoid tissue. Arthritis Rheum.

[9] Nanagara R, Duray PH, Schumacher HR (1996). Ultrastructural demonstration of spirochetal antigens in synovial fluid and synovial membrane in chronic Lyme disease: possible factors contributing to persistence of organisms. Hum Pathol.

[10] Crossland NA, Alvarez X, Embers ME (2018). Late disseminated Lyme disease: associated pathology and spirochete persistence posttreatment in rhesus macaques. Am J Pathol.

[11] Parthasarathy G, Fevrier HB, Philipp MT (2013). Non-viable Borrelia burgdorferi induce inflammatory mediators and apoptosis in human oligodendrocytes. Neurosci Lett.

[12] Greenmyer JR, Gaultney RA, Brissette CA, Watt JA (2018). Primary human microglia are phagocytically active and respond to Borrelia burgdorferi with upregulation of chemokines and cytokines. Front Microbiol.

[13] Chen MK, Guilarte TR (2008). Translocator protein 18 kDa (TSPO): molecular sensor of brain injury and repair. Pharmacol Ther.

[14] Coughlin JM, Wang Y, Ma S, Yue C, Kim PK, Adams AV (2014). Regional brain distribution of translocator protein using [^11^C]DPA-713 PET in individuals infected with HIV. J Neuro-Oncol.

[15] Wang Y, Coughlin JM, Ma S, Endres CJ, Kassiou M, Sawa A (2017). Neuroimaging of translocator protein in patients with systemic lupus erythematosus: a pilot study using [^11^C]DPA-713 positron emission tomography. Lupus.

[16] Coughlin JM, Wang Y, Munro CA, Ma S, Yue C, Chen S (2015). Neuroinflammation and brain atrophy in former NFL players: an in vivo multimodal imaging pilot study. Neurobiol Dis.

[17] Kobayashi M, Jiang T, Telu S, Zoghbi SS, Gunn RN, Rabiner EA (2018). ^11^C-DPA-713 has much greater specific binding to translocator protein 18 kDa (TSPO) in human brain than ^11^C-(*R*)-PK11195. J Cereb Blood Flow Metab.

[18] CDC. Lyme Disease (Borrelia burgdorferi) 2011 Case Definition. In: National Notifiable Diseases Surveillance System Surveillance Case Definitions: Lyme Disease 2011. https://wwwn.cdc.gov/nndss/conditions/lyme-disease/case-definition/2011/. Accessed 15 Oct 2018.

[19] Martin A, Boisgard R, Kassiou M, Dolle F, Tavitian B (2011). Reduced PBR/TSPO expression after minocycline treatment in a rat model of focal cerebral ischemia: a PET study using [^18^F]DPA-714. Mol Imaging Biol.

[20] Touradji P, Aucott JN, Yang T, Rebman AW, Bechtold KT. Cognitive decline in post-treatment Lyme disease syndrome. Arch Clin Neuropsychol. 2018. 10.1093/arclin/acy051. 10.1093/arclin/acy051 29945190

[21] Thominiaux C, Dolle F, James ML, Bramoulle Y, Boutin H, Besret L (2006). Improved synthesis of the peripheral benzodiazepine receptor ligand [^11^C]DPA-713 using [^11^C]methyl triflate. Appl Radiat Isot.

[22] Endres CJ, Pomper MG, James M, Uzuner O, Hammoud DA, Watkins CC (2009). Initial evaluation of ^11^C-DPA-713, a novel TSPO PET ligand, in humans. J Nucl Med.

[23] Buckner RL, Head D, Parker J, Fotenos AF, Marcus D, Morris JC (2004). A unified approach for morphometric and functional data analysis in young, old, and demented adults using automated atlas-based head size normalization: reliability and validation against manual measurement of total intracranial volume. NeuroImage.

[24] Innis RB, Cunningham VJ, Delforge J, Fujita M, Gjedde A, Gunn RN (2007). Consensus nomenclature for in vivo imaging of reversibly binding radioligands. J Cereb Blood Flow Metab.

[25] Eckman EA, Pacheco-Quinto J, Herdt AR, Halperin JJ (2018). Neuroimmunomodulators in neuroborreliosis and Lyme encephalopathy. Clin Infect Dis.

[26] Notter T, Coughlin JM, Sawa A, Meyer U (2018). Reconceptualization of translocator protein as a biomarker of neuroinflammation in psychiatry. Mol Psychiatry.

[27] Baecher-Allan C, Kaskow BJ, Weiner HL (2018). Multiple sclerosis: mechanisms and immunotherapy. Neuron.

[28] Army Individual Test Battery: Manual of directions and scoring. 1944;

[29] Benedict RHB, Schretlen D, Groninger L, Brandt J (1998). Hopkins verbal learning test – revised: normative data and analysis of inter-form and test-retest reliability. Clin Neuropsychol.

[30] Wechsler D (2008). Wechsler Adult Intelligence Scale, 4th edition.

[31] Stuss DT, Stethem LL, Poirier CA (1987). Comparison of three tests of attention and rapid information processing across six age groups. Clin Neuropsychol.

