# Supplementary figures and images for: Imaging glial activation in patients with post-treatment Lyme disease symptoms: a pilot study using [11C]DPA-713 PET
Source: J Neuroinflammation. 2018 Dec 19;15:346. doi: 10.1186/s12974-018-1381-4 (PMC6299943; doi:10.1186/s12974-018-1381-4)

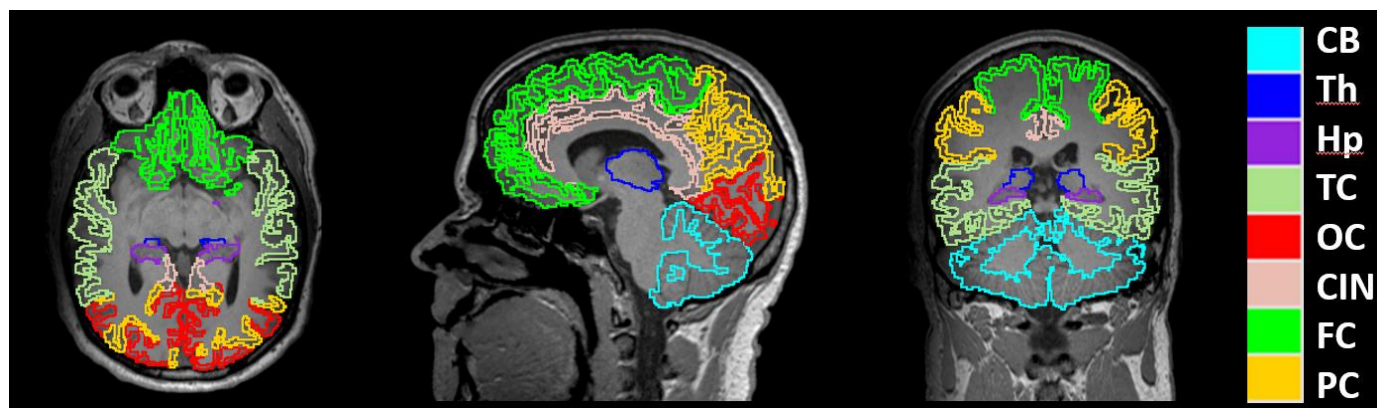

Supplement: Supplementary file 1 — Figure S1. Representative volumetric segmentation demonstrating the eight regions of interest. Abbreviations: CB, cerebellum; Th, thalamus; Hp, hippocampus; TC, temporal cortex; OC, occipital cortex; CIN, cingulate cortex; FC, frontal cortex; PC, parietal cortex. (PDF 231 kb) [file 12974_2018_1381_MOESM1_ESM.pdf]

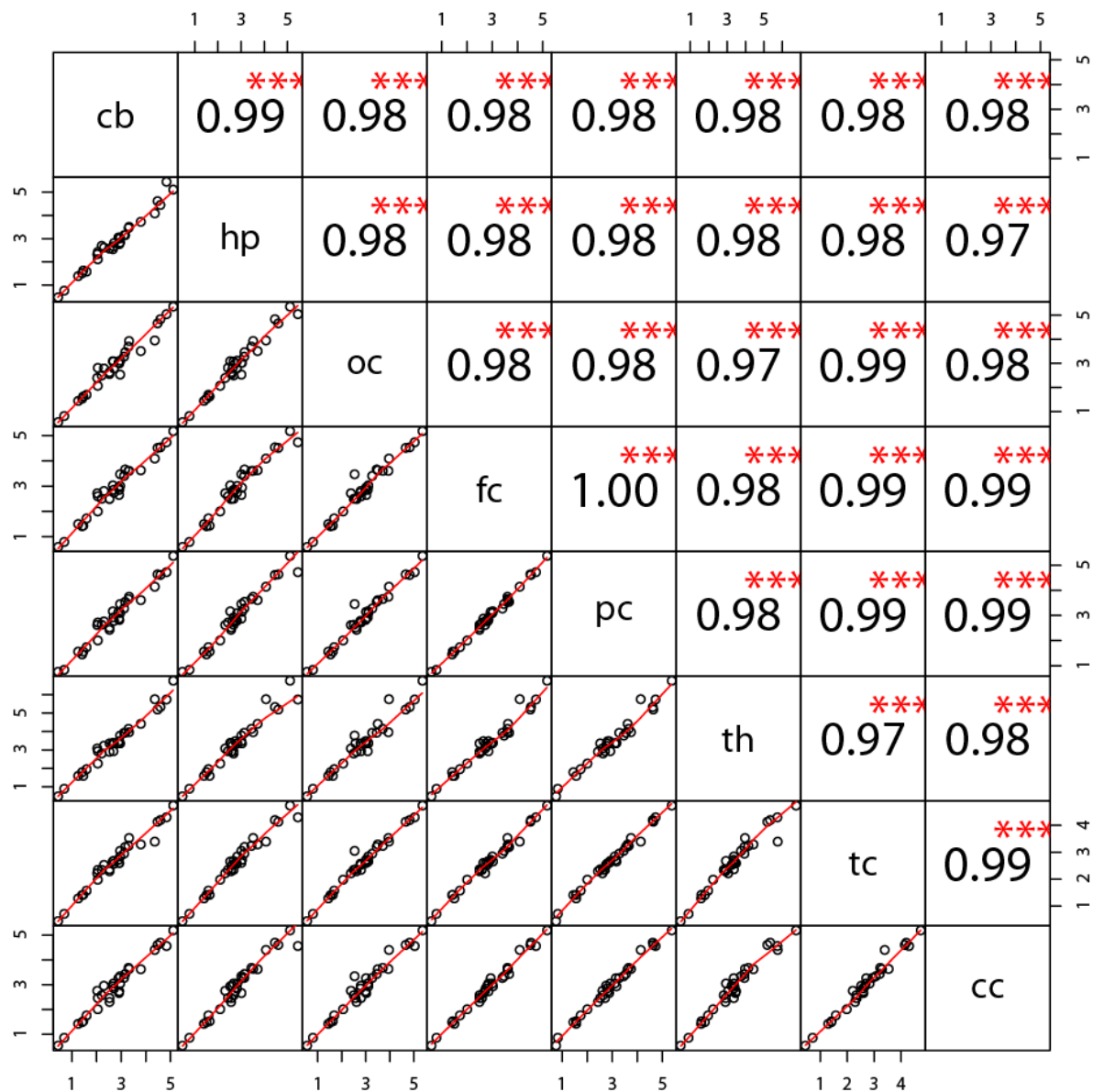

Supplement: Supplementary file 2 — Figure S2. Correlation matrix using [11C]DPA-713 binding data from the eight regions of interest. All correlation coefficients are greater than or equal to 0.97 (indicating very strong correlations). Abbreviations: cb, cerebellum; hp., hippocampus; oc, occipital cortex; fc, frontal cortex; pc, parietal cortex; th, thalamus, tc, temporal cortex; cc, cingulate cortex. (PDF 233 kb) [file 12974_2018_1381_MOESM2_ESM.pdf]
